# Feline dystocia and kitten mortality up to 12 weeks in pedigree cats

**DOI:** 10.1177/1098612X241284766

**Published:** 2024-12-10

**Authors:** Petra Černá, Sneha Joseph Pugalendhi, Darren J Shaw, Danièlle A Gunn-Moore

**Affiliations:** 1 Department of Clinical Sciences, Colorado State University, Fort Collins, CO, USA; 2 Small Animal Clinic, The University of Veterinary Sciences Brno, Brno, Czech Republic; 3 The Royal (Dick) School of Veterinary Studies and The Roslin Institute, University of Edinburgh, Edinburgh, UK

**Keywords:** Breeders, birth defects, pregnancy, kittens

## Abstract

**Objectives:**

The aim of the present study was to establish the incidence of dystocia in pedigree cat breeds and investigate mortality rates in kittens up to the age of 12 weeks.

**Methods:**

A retrospective study was conducted that utilised convenience sampling. Registered cat breeders from 29 countries whose cats had given birth within a single year (2019) were asked to complete an online questionnaire. This study is the third in a series over 30 years that have assessed changes in birth-related statistics in cat breeds.

**Results:**

Data were collected from 448 breeders on 853 litters, with 3560 live-born kittens from 45 pedigree breeds (as per the queens’ breed). The incidence of dystocia that required veterinary intervention was 14.9% and varied by breed (range 0–22.2%). Caesarean section was required for 10.7% of litters, most commonly in Cornish Rex cats. Despite apparent breed variation, there was no statistically significant difference among breeds. Queens aged over 5 years and litters of more than six kittens were associated with a reduced risk of dystocia, whereas a longer gestation period increased the risk. Bengal cats had the highest cumulative kitten mortality up to the age of 12 weeks (23.4%). Significant breed differences (*P* <0.001) were noted, with Ragdoll and Norwegian Forest Cats having lower litter mortality compared with Bengal and British Shorthair/Longhair cats.

**Conclusions and relevance:**

The incidence of dystocia in pedigree cats was higher than the historical values for non-pedigree and pedigree cats. The age of the queen, gestation length and litter size affected the incidence of dystocia. Kitten mortality was affected by the requirement for caesarean section, presence of birth defects and breed.

## Introduction

Dystocia is a recognised cause of neonatal mortality and stillbirth in pet cats (*Felis catus*), and pedigree cats have been reported as having a higher prevalence of dystocia compared with their non-pedigree counterparts.^1^

Dystocia occurs as a result of either functional causes (ie, uterine inertia) or obstructive causes (eg, maternal and fetal factors). Functional dystocia is seen when the myometrium of the uterus fails to produce sufficient contractions to expel the kittens through the birth canal. It can be either primary or secondary, with the most common cause in cats being primary uterine inertia.^2^ Primary uterine inertia occurs when the uterus fails to contract effectively from the start of labour, whereas secondary uterine inertia occurs when the uterus initially contracts effectively, but then weakens or stops contracting altogether during the course of labour.^2^ Obstructive dystocia may be attributed to maternal factors (which occurred in 69% of litters in a recent study) and fetal factors (which occurred in 31% of litters), or a combination of the two.^3^ Obstructive dystocia typically occurs as a result of fetal malpresentation and malformations, and is associated with litter size (both small and large litters).^1,4–6^

Dystocia increases the risk of fetal death by hypoxia.^1^ Dystocia is managed through three primary methods: medical intervention, such as administering oxytocin to strengthen uterine contractions and administering glucose or calcium where a deficit has been noted; non-surgical manipulation, such as fetal manipulation; or surgical intervention by performing a caesarean section (c-section).^3,7^ Dystocia has been reported to occur in 0.4–8% of all cat litters, occurring more frequently in pedigree cats than non-pedigree cats.^1,6^ A 2017 Swedish study reported the incidence of dystocia as 22 cats per 10,000 cat-years at risk, with a nearly 10 times higher risk in purebred cats compared with domestic shorthair cats.^8^

Pet ownership has reached an all-time high in the UK.^9^ There were 11 million pet cats in the UK in 2022, with pedigree cats increasing from 17% more than 5 years ago to 45% in 2024.^9^ With the increasing demand for pedigree cats in the UK, it remains vital to update our understanding of how often dystocia occurs and what factors may affect it. This study was the third in a series of studies that have run over 30 years.^1,5^ The aims of this study were to establish the current incidence of dystocia in different pedigree cat breeds and investigate mortality rates in litters of different breeds in their first 12 weeks of life.

## Materials and methods

A questionnaire was designed and distributed online through the survey platform JISC Surveys (available at https://app.onlinesurveys.jisc.ac.uk). It was promoted on the International Cat Care website and by social media (eg, Facebook groups for cat breeders) and distributed to known breeders. It was open from 10 April 2020 to 31 December 2021. Participants had to be aged at least 18 years and registered with a feline breeding organisation. All answers were anonymous. Ethical approval was granted from the Royal (Dick) School of Veterinary Studies’ Human Ethical Review Committee.

The questionnaire was based on the 2006 study by Sparkes et al.^5^ It asked questions about the following: (1) the breeding queens; (2) the birth of kittens; and (3) the kittens’ first 12 weeks of life (Appendix 1). Participants were asked how many litters had been born in 2019 (so that recent memory of a full year would be assessed) and answered the same questions for each litter.

### Statistical analysis

Responses were grouped by the breed of the queen for each litter, with variables of interest about the birth recorded for each queen, plus the queen’s age, gestation length, litter size, kitten mortality percentage and number of litters with birth defects. Sister breeds (Abyssinian and Somali; Exotic Shorthair and Persian; Balinese, Siamese, Oriental and Havana; Russian Blue and Nebelung) were combined for statistical analyses. The c-section rate was used as a direct marker for dystocia.

Statistical analyses were performed using R (2023, The R Foundation for Statistical Computing). Generalised linear models with binomial errors (simple linear and polynomial fits, as appropriate) were performed for the need for a c-section, occurrence of birth defects and litter mortality, with odds ratios (ORs) and associated 95% confidence intervals (CIs) calculated. Where no adequate fit was obtained, locally estimated scatterplot smoothing (LOESS) running averages are presented. Only breeds with at least 10 litters (n = 18 breeds) were analysed to reduce the impact of those with breeds with few litters. Pairwise post-hoc Tukey comparisons were undertaken to assess the differences between breeds. A *P* value <0.05 was considered statistically significant.

## Results

**Figure 1:**
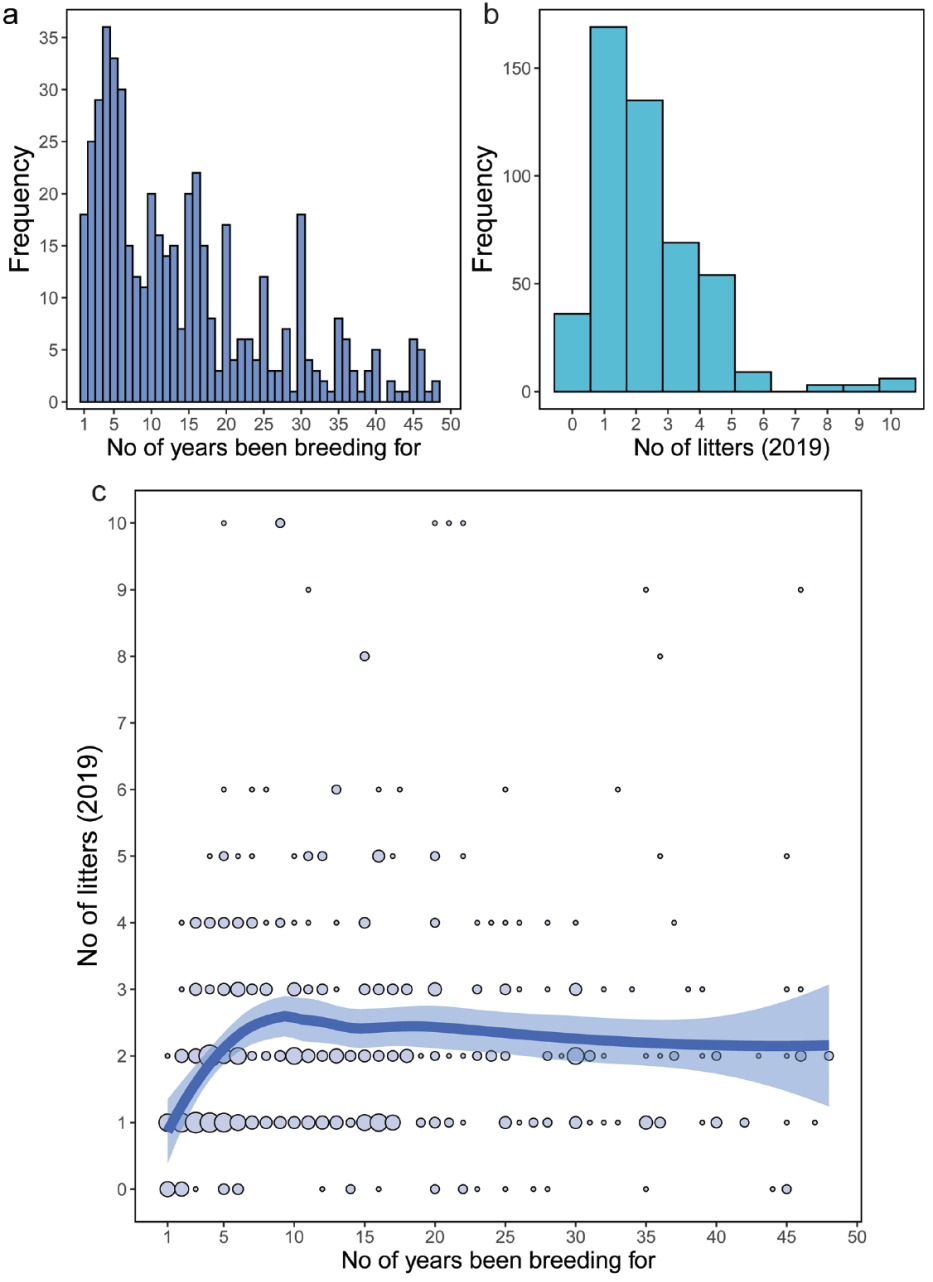
(a) Histogram showing the frequency of the number of years breeders had been breeding cats; (b) histogram showing the frequency of the number of litters born in 2019; (c) scatterplot showing the number of litters born in 2019 against the number of years breeders had been breeding cats. The size of the points indicates the number of responses for a particular combination. The thick line is a LOESS running average, with the shaded area representing the standard error. LOESS = locally estimated scatterplot smoothing

A total of 484 responses were received, with 27.9% of breeders based in the UK. The rest came from outside the UK, of which 9.5% were from the USA. Most breeders were registered with Fédération Internationale Féline (FIFe; 53.9%) and had considerable experience in breeding cats, with 37.8% having bred pedigree cats for more than 15 years (mean 15, range 1–48) (Figure 1a).

Almost all of the breeders (n = 448, 99%) reported kitten births in 2019, with 892 litters delivered. There was a maximum of 10 litters per breeder (Figure 1b). The 36 breeders who did not have a litter born in 2019 were excluded from further analysis. For the rest, 37.7% reported a single litter (Figure 1b), with an overall mean of 2.34 litters. The number of litters per queen varied according to breeder experience, increasing up to 10 years’ experience, after which it plateaued (Figure 1c).

Detailed information on litter composition was provided for 853 litters, with 3560 live-born kittens from 45 pedigree breeds (as per the queens’ breed); 39 litters were excluded because of incomplete information. In total, 18 breeds produced ⩾10 litters, resulting in 768 litters with 3215 kittens (Table 1). Ragdoll, Oriental group and Cornish Rex cats had the longest gestation periods, whereas Korats, Sacred Birmans and Bengals had the shortest. Burmese cats had the largest litters, whereas Abyssinian/Somalis had the smallest, with the overall mean litter size being 4.5 kittens.

**Table 1:** Summary of pedigree breeds with at least 10 litters for each breed, including the number of litters, mean age of the queens, mean gestation length, mean litter size, kitten mortality percentage, percentage of litters with obvious birth defects and percentage of c-sections

Breed	Litters (n)	Mean age of the queen (years)	Mean gestation length (days)	Mean litter size (n)	Mean kitten mortality (%)	Litters with defects (%)	C-section rate (%)
Abyssinian/Somali	55	3.02	65.27	3.49	10.9	12.7	9.1
Balinese/ Siamese/Oriental/Havana	70	2.99	66.15	4.83	17.8	11.4	20.0
Bengal	19	3.32	63.95	4.95	23.4	5.3	10.5
BSH/BLH	160	3.61	65.16	4.88	18.1	13.1	13.1
Burmese	24	3.67	64.25	5.25	16.7	12.5	20.8
Cornish Rex	18	3.44	65.83	4.67	7.1	5.6	22.2
Devon Rex	31	3.13	64.26	3.90	12.4	22.6	9.7
Exotic/Persian	44	4.34	65.23	3.34	15.6	4.5	2.3
Korat	10	4.70	63.3	4.50	8.9	20.0	0
Maine Coon	95	3.75	65.38	5.17	13.0	12.6	10.5
Neva Masquerade	16	2.88	65.31	4.62	10.8	6.2	0
Norwegian Forest Cat	65	4.40	65.00	5.05	9.5	10.8	10.8
Ragdoll	39	3.38	66.22	4.87	7.4	2.6	2.6
Russian Blue/Nebelung	20	4.05	64.56	4.10	13.4	10.0	10.0
Sacred Birman	44	3.84	63.57	3.59	19.0	11.4	9.1
Selkirk Rex (Short/Long)	18	3.11	65.39	4.06	15.1	5.6	11.1
Siberian	26	3.65	64.19	4.50	7.7	7.7	11.5
Sphynx	14	4.43	64.93	4.57	21.9	21.4	14.3
Mean	42.67	3.65	64.89	4.46	13.81	10.89	10.19

Sister breeds were combined for the purposes of this study (ie, Abyssinian/Somali, Balinese/Siamese/Oriental/Havana and Russian Blue/Nebelung). BSH and BLH were by far the most prevalent breeds, followed by Maine Coon cats

BLH = British Longhair; BSH = British Shorthair; c-section = caesarean section

### Veterinary and breeder assistance

**Figure 2:**
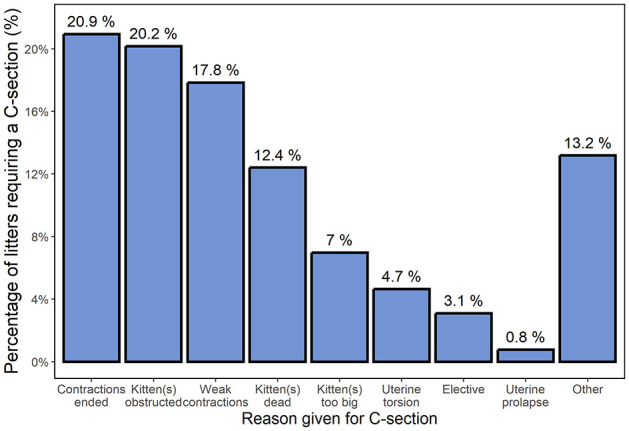
Bar graph summarising the different reasons for cats requiring a c-section

More than one-third of litters (292/853, 34.2%) were reported as requiring veterinary or breeder assistance during delivery. Where veterinary help was needed, this involved fetal manipulation and/or medication (eg, oxytocin), with or without c-section. Veterinary assistance was reported for 127 (14.9%) litters, with the majority of these (91/127, 71.7%) needing a c-section (ie, 91/853 [10.7%] of all litters) (Table 1). Over 40% of c-sections were a result of contractions ending (functional dystocia, 20.9%) and kitten(s) obstructing further labour (obstructive dystocia, 20.2%) (Figure 2); few (3.1%) were elective.

**Figure 3:**
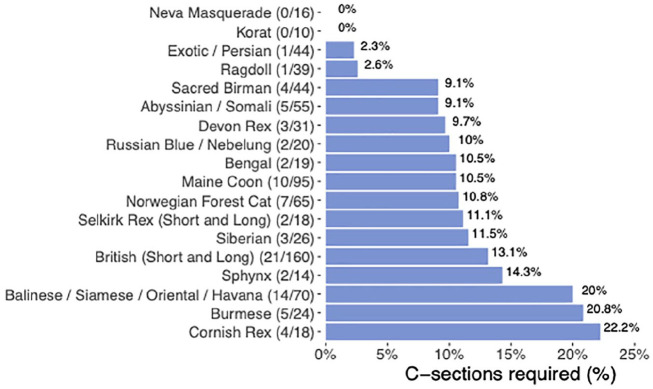
Bar graph of the prevalence of c-section plotted against breed (for the 18 breeds with at least 10 litters born). (n/nn) n = number of c-sections for breed, nn = total number of litters for a particular breed

The highest percentage of c-sections (22.2%, 4/18) was seen in the Cornish Rex cats, followed by Burmese (20.8%, 5/24) and Oriental group cats (20%, 14/70) (Figure 3). In contrast, Korats and Neva Masquerades had no c-sections, while Exotic/Persians (1/44) and Ragdolls (1/39) required c-sections in fewer than 3% of litters. Despite apparent breed variation in the requirement for c-section, there were no statistically significant differences (*P* = 0.099).

**Figure 4:**
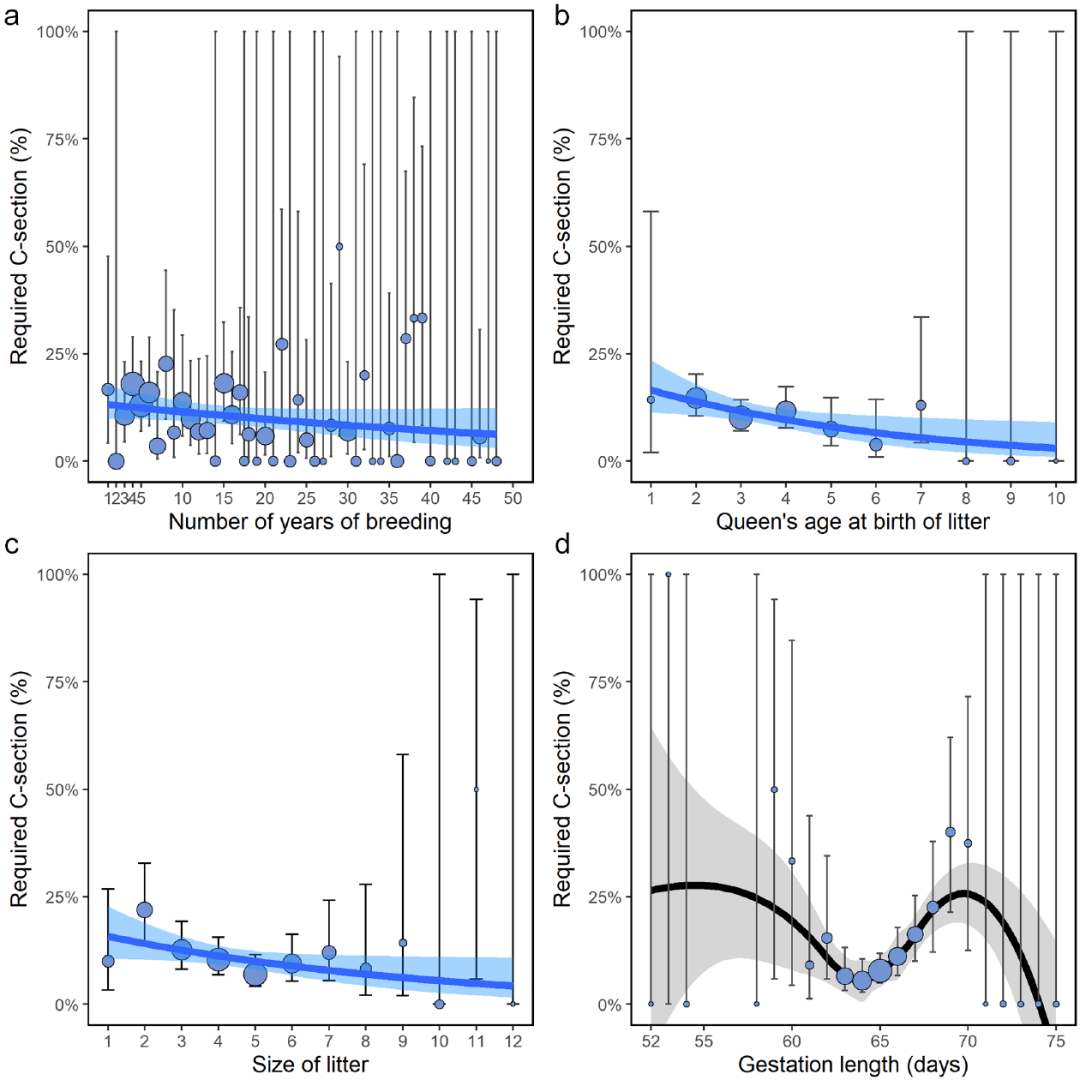
Relationship between requirement for c-section and (a) number of years a breeder has been breeding; (b) age of queen at birth of litter; (c) size of litter; and (d) gestation length. Size of points are scaled to the number of litters at a particular x-value. Vertical lines are the 95% confidence intervals for each prevalence value. (a–c) Solid lines and associated shaded areas represent the fitted linear binomial generalised linear model fit and standard errors. (d) Solid line and associated shaded area represent a LOESS running average and standard error. LOESS = locally estimated scatterplot smoothing

Several factors were found to affect the requirement for a c-section. Increased years of breeder experience were not associated with a decreased need for c-section (OR 0.98, 95% CI 0.96–1.00) (Figure 4a). However, older queens appeared to have a reduced risk (OR 0.81, 95% CI 0.68–0.96; *P* = 0.017) (Figure 4b); a c-section was needed in 12/186 (<6.5%) queens aged ⩾5 years (see below), compared with 31/211 (15%) queens aged <3 years. Larger litters also appeared to have a reduced risk (OR 0.88, 95% CI 0.77–0.99; *P* = 0.042) (Figure 4c); a c-section was needed in only 10/100 (10%) litters with more than six kittens, compared with 19/103 (18%) litters with fewer than three kittens. This compares with 34/392 (8.7%) litters of four or five kittens that required a c-section (this is of interest as the mean litter size overall was 4.5 kittens). In contrast to the linear declines associated with older queens and larger litters, there was a more complicated, non-linear relationship between increased risk of c-section and gestation length. Risk decreased to a minimum of 64 days before increasing again as gestation increased to 70 days (Figure 4d).

### Birth defects

**Figure 5:**
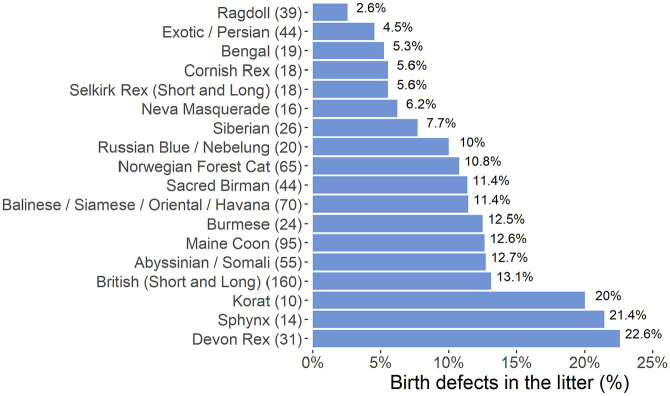
Bar graph of the prevalence of birth defects per litter plotted against the 18 breeds that had at least 10 litters born in 2019. Values in parentheses indicate number of litters for the particular breed

**Figure 6:**
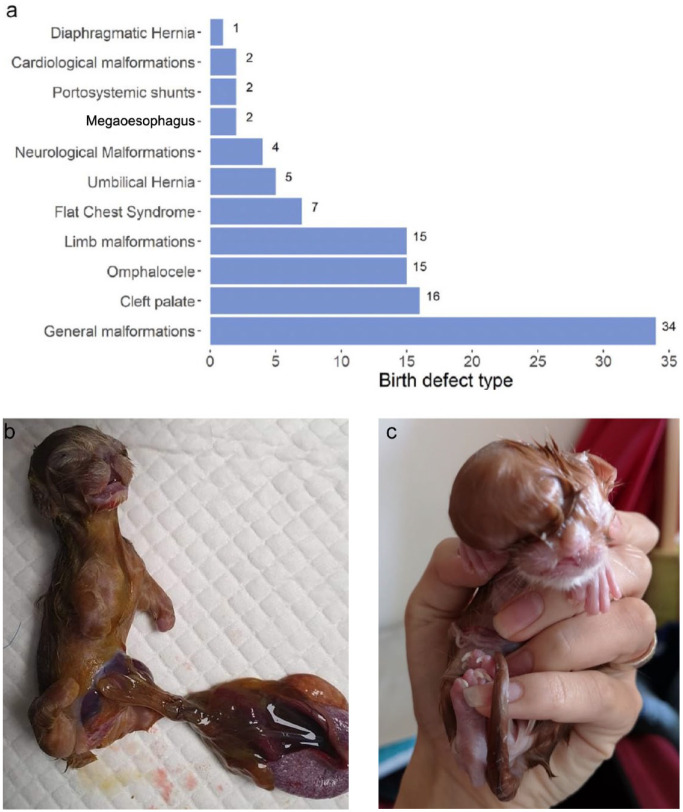
(a) Bar graph summarising the frequency of different birth defects. (b,c) Birth defects in two kittens: (b) a midline defect, omphalocele and limb deformity and (c) a cranial defect

Birth defects were reported in 10.9% of 853 litters. They occurred most commonly in Devon Rex and Sphynx cats (>20%) and least in Ragdoll and Exotic/Persian cats (<5%) (Figure 5). Despite this, there were no significant differences among breeds for the presence of birth defects (*P* = 0.549). Descriptions of defects were given for 102 litters (Figure 6a); 32 (31.4%) kittens were reported to have general malformations (eg, incomplete fetal development, mummification), with the most common specific malformations being cleft palate (n = 16, 15.7%), omphalocele (n = 15, 14.7%) and limb malformations (n = 15, 14.7%) (Figure 6b,c).

The main factor associated with birth defects was increased litter size (OR 1.19, 95% CI 1.06–1.32; *P* = 0.002) (see Figure 1a in the supplementary material), with birth defects increasing from less than 9% in litters with fewer than five kittens to 14% in litters with more than six kittens. The relationship between birth defects and gestation was non-linear, with a general decline to 6% at a gestation length of 66 days, before rising to a second peak of 20% at a gestation length of 69 days (see Figure 1b in the supplementary material). The age of the queen and the years of a breeder’s experience were not associated with birth defects (*P* >0.066) (see Figure 1c,d in the supplementary material).

### Litter mortality

**Figure 7:**
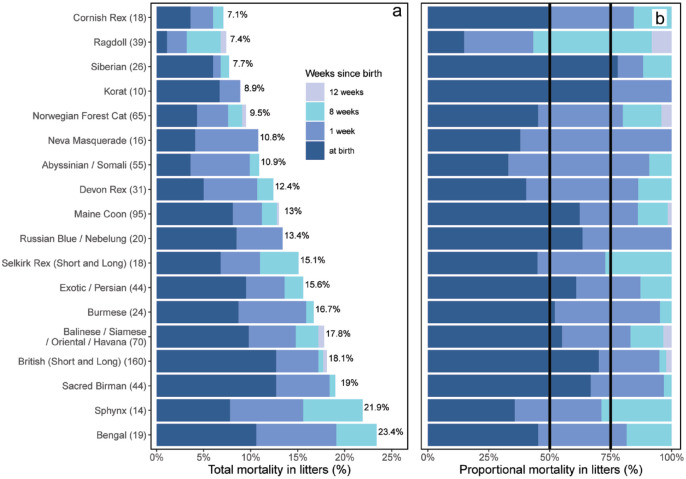
(a) Bar graph of cumulative percentage mortality of kittens in the first 12 weeks of life (for the 18 breeds with at least 10 litters born in 2019). (b) Stacked bar graph of proportional percentage mortality of kittens in the first 12 weeks of life (for the18 breeds with at least 10 litters born in 2019). Vertical lines indicate 50% and 75% mortalities. Values in parentheses indicate number of litters for the particular breed

The highest mortality in kittens aged up to 12 weeks (stillbirths included) was for the Bengal breed (23.4%, 95% CI 17.0–33.58), followed by the Sphynx (21.9%, 95% CI 13.4–33.6) and Sacred Birman (19.0%, 95% CI 13.6–25.9) (Figure 7). Cornish Rex cats had the lowest kitten mortality (7.1%, 95% CI 3.2–15.0), followed by Ragdolls (7.4%, 95% CI 4.4–12.1). There were statistically significant differences in litter mortality among the breeds (*P* <0.001), with Ragdoll and Norwegian Forest Cats having lower rates than Bengal and British Shorthair and Longhair (BSH/BLH) cats.

The age at death varied among the breeds. For three breeds (Korat, Neva Masquerade and Russian Blue) (Figure 7a,b), kitten deaths occurred only at birth and in the first week of life. With the exception of Ragdolls (43.2%), more than 70% of kitten deaths in all breeds occurred at birth and in the first week (Figure 7a). Only five breeds (Ragdoll, Norwegian Forest Cat, Maine Coon, BSH/BLH and Oriental group cats) had litters where additional mortality occurred between 8 and 12 weeks of age. For 10/18 of the most common breeds, more than 50% of mortalities were observed at birth (Figure 7b).

Lower kitten mortality up to the age of 12 weeks was associated with increased queen age (OR 0.90, 95% CI 0.84–0.96; *P* = 0.001) (see Figure 2a in the supplementary material). The majority of kittens (96%) survived to 12 weeks of age if they were born to queens aged over 8 years compared with an 85% survival in kittens born to queens aged under 4 years. There was a more complicated polynomial fit for litter survival with gestation length (*P* <0.001) (see Figure 2b in the supplementary material). Survival increased rapidly from short (<57 days) gestation lengths, plateauing to over 80% if the gestation length was more than 61 days, with an apparently higher plateau of over 93% if the gestation length extended to more than 69 days (though this only represented 19 litters). Greater mortality by the age of 12 weeks was associated with c-section (OR 3.43, 95% CI 2.70–4.34; *P* <0.001). The survival rate of kittens living to 12 weeks in queens who needed a c-section was lower (69%) than the survival rate of kittens living to 12 weeks in queens who did not require a c-section (88%). A similar pattern was observed for birth defects (OR 2.79, 95% CI 2.22–3.49; *P* <0.001), with the survival rate of kittens living to 12 weeks in litters with birth defects (73%) being lower than in litters without birth defects (88%). Increasing litter size was associated with a polynomial change in mortality (*P* = 0.003) (see Figure 2c in the supplementary material), with an increasing percentage of kittens surviving to 12 weeks in queens as litter size increased, peaking at 91% for a litter of five kittens, before declining to less than 75% as litter size increased further. In contrast, increasing breeder experience was not associated with decreased litter mortality (OR 1.00, 95% CI 0.99–1.02; *P* = 0.242) (see Figure 2d in the supplementary material).

## Discussion

This study is the third in a series of three questionnaire-based surveys that have been conducted over 30 years to assess the prevalence of dystocia, requirement for c-sections and mortality in kittens up to the age of 12 weeks across different pedigree cat breeds. In the current study, 10.2% of litters required a c-section. This has increased compared with 5.8% for non-pedigree cats and 7–10% for pedigree cats in 1995,^1^ and 8.0% for pedigree cats in 2006.^5^ The prevalence of dystocia varied among breeds, though not significantly. This could have resulted from small numbers of litters in certain breeds. Of the 18 breeds with at least 10 litters, only the BSH/BLH cats had more than 100 litters (Table 1). Perhaps unexpectedly, other studies have also found breed not to affect dystocia rates.^5,6^ This may be because other, non-breed related factors, such as litter size, queen age and gestation length, have a bigger impact on dystocia rates.^5^ Obesity has also been shown to influence rates of dystocia, and has been linked with the rate of stillbirths;^8,10^ however, the body condition score of queens was not tracked in the current study. In contrast, another study found breed to be a significant risk factor for c-section, having found differences in incidence rate ratios (IRRs) among breeds, with BSH/BLH and Oriental cats having increased IRRs compared with other breeds.^8^ Different breeds may have different risks of dystocia owing to their head and/or body shape, or as a result of non-phenotypic genetic changes.^2,8^ For example, brachycephalic queens may have a higher risk of dystocia and/or c-sections owing to changes in pelvic conformation.^11^

This study found that queens aged over 5 years had a decreased risk of requiring a c-section. It is possible that older queens who had previously given birth to healthy kittens with no complications were bred from preferentially. This supports an older study that suggested it is possible to breed for a low level of dystocia by selectively breeding from queens who previously bred easily.^1^ In contrast, younger queens who experienced dystocia or birth defects were likely to be retired from breeding. Another study found a decreased risk of c-section with increasing parity, which roughly corresponds with increasing queen age.^5^

Litter size was also found to affect the incidence of dystocia, with litters of one or two kittens having an increased incidence of dystocia compared with litters with a mean size of 4.5 (15% vs 8.7%, respectively). Though litter sizes of more than six kittens appeared to have a decreased incidence of dystocia, care needs to be taken in interpreting this, given smaller sample sizes. In contrast, a previous study found increased litter sizes (more than six kittens) had an increased risk of dystocia (eg, 34% of litters with nine kittens experienced dystocia vs 5% of litters with six kittens).^6^ That study also found that litters of five or six kittens had the least dystocia (approximately 5%), with single-kitten litters also having an increased risk (12.5%),^6^ similar to the findings of the current study. Very small and very large litters can result in dystocia due to under- or overdistension of the uterus, respectively; in addition, overly large kittens are most likely to occur in small litters and these kittens are at risk of dystocia from fetal–maternal size mismatch.^1,6,8^ Although previous studies found that mean kitten size relates to the number of kittens in the litter,^6,8^ this was not investigated in the current study as kitten birth weights were not collected. Further studies are needed to investigate the association between kitten size, litter size and the effect on dystocia to clarify this relationship.

The Exotic/Persian cats had one of the lowest rates of dystocia in the current study (2.3%), consistent with another study that found a low IRR for dystocia for these breeds.^8^ However, the decrease in dystocia (from 7.5%)^1^ occurred while the brachycephalic features, such as the reduction of facial bones and deformation of the neurocranium, have become more pronounced.^10^ This was unexpected, as the increasing severity of brachycephalic features would be expected to predict more dystocia owing to the decrease in the wedge effect of the kitten’s nose, which is believed to be necessary to engage the maternal pelvis to stimulate labour.^1,10^ The current study also found the cumulative kitten mortality of Exotic/Persian cats to be markedly lower than in a previous study (15.6% vs 25%).^5^

One possible reason behind the reduced dystocia and kitten mortality in Exotic/Persian cats could be the near elimination of autosomal dominant polycystic kidney disease (PKD) in the breed.^12^ Targeted intervention (eg, by the Governing Council of the Cat Fancy and FIFe) mandating that breeding queens be genetically tested for the PKD gene defect (*PKD1*) has decreased the prevalence of PKD in Exotic/Persian cats from 28% in 2005 to 2% in 2016.^12^ Newer studies have suggested that PKD in cats may have genetic heterogeneity, as seen in people, and have implicated the *PKD2*, *DZIP1L* and *PKDH1* genes.^13,14^ Moreover, the genes responsible for PKD in humans have also been demonstrated to show pleiotropy, influencing multiple biological processes, such as the vascular and skeletal system, beyond just causing PKD.^15^ It may be that the targeted intervention in reducing PKD in the breed may have indirectly selected for a fitter breeding population, leading to a decrease in the rates of dystocia and cumulative kitten mortality given the likely pleiotropic nature of the genes involved. It is still unclear what role, if any, reducing the prevalence of PKD has played in reducing the incidence of dystocia and/or overall kitten mortality in this breed. More research is needed to determine how pleiotropic PKD-relevant genes are in Exotic/Persian cats and what effects they may have on overall reproductive fitness.

Birth defects were found in 10.9% of litters, which was lower than in 2006 (14.3%).^5^ Litter size was linked to birth defects in both studies, with larger litters being associated with more birth defects and stillbirths.^5,6^ Increased numbers of birth defects in large litters could result from poorer kitten development in utero due to less space within the uterus and/or nutritional deficiencies.^16^ Although breed was found not to be a significant factor for birth defects in the current study, a previous study has postulated that congenital defects are heritable and breed related, though further research is needed to look into this.^6^ It has been reported that litter sizes vary among breeds,^5^ and if certain breeds are more prone to having larger litters, they may (potentially) be at a higher risk of more birth defects due to the increased litter size. More research is needed to understand the complex nature of the different variables involved and whether there are confounding or correlative effects.

The current study found that kitten mortality up to 12 weeks of age was affected by the queen’s breed and age, gestation length, delivery by c-section and the presence of birth defects. Ragdolls and Norwegian Forest Cats had statistically lower kitten mortality compared with Bengal and BSH/BLH cats; however, why this should be the case is currently unclear. Breed variations have been previously demonstrated to be a factor in kitten mortality, with Oriental Shorthair/Siamese and Exotic/Persian cats having a much higher rate than other pedigree breeds (>10%).^6^ Kitten mortality was reduced in queens aged under 8 years, as has previously been suggested, whereas older queens may be prone to higher rates of kitten mortality and stillborn kitten rates.^6^ A gestation length of more than 61 days was also shown to decrease kitten mortality. Gestations of ‘species appropriate length’ usually result in the kittens being born fully developed and strong enough to suckle efficiently; unsurprisingly, kittens born at the extremes of the range have a lower chance of being viable.^5^ Although it is possible that some specific cat breeds have gestation lengths slightly longer or shorter than most cats, this has not yet been reported.

Birth defects and c-sections were associated with high kitten mortality up to 12 weeks of age. A recent study where queens were presented to a veterinary hospital for dystocia reported that overall neonatal survival to discharge was as low as 66%, and that dystocia led to increased kitten mortality.^3^ Another study reported lower vitality scores in kittens delivered by emergency c-section compared with those delivered naturally.^16^ Postoperative pain and stress associated with a c-section may result in the queen being reluctant to suckle her kittens; it may also reduce colostrum flow, preventing the kittens from suckling immediately and leading to failure in passive transfer of immunity to the kittens. Colostrum-deprived kittens are shown to have lower levels of immunoglobulins even when supplemented with milk replacers or fostered onto surrogate mothers.^18^ This can lead to a greater risk of infection during this period, which, in turn, can result in higher kitten mortality. In the current study, most neonatal deaths occurred in the first week of life, as has been reported previously.^5,8^ Most deaths in week 1 of life are unlikely to result from infectious disease as colostrum should protect kittens in the first 4 weeks of life; however, the duration of maternally derived immunity can be variable.^19^ Kitten deaths in the first week of life are more often related to dystocia-related hypoxia and trauma.^20^ Although a previous study reported that non-pedigree kittens were more likely to have a diagnosis of feline panleukopenia than pedigree kittens,^6^ factors that may impact kitten mortality as a result of infectious diseases were not within the scope of this study; a larger study population would need to include a non-pedigree population for comparison.

The present study has some limitations. The main limitations were the convenience sampling method utilised (vs a true randomised prevalence study) and the retrospective nature of the study. Another limitation was that although over 800 queens were included, the number of queens within each breed was sometimes limited. Respondents were only asked the kittens’ breed; this made it difficult to ascertain the queen’s breed when breeders had several litters from queens of different breeds. The queen’s breed had to be inferred from the kitten’s breed and survey responses were excluded where this was not possible. In addition, not all breeds were equally represented, which may have led to values for some breeds being unrepresentative of the true population; however, the same can be said of other similar studies.^1,5^

## Conclusions

This study is the third to be conducted over a period of more than 30 years that details the changing face of feline dystocia and kitten mortality. It shows that dystocia is more common in pedigree cats than seen historically; however, significant improvements have occurred in certain breeds, specifically the Persian/Exotic breed. The rates of dystocia varied among breeds, although breed was not a significant factor affecting this. Instead, non-breed factors, such as age, litter size and gestation length, have a significant impact on the rates of dystocia. Litter mortality up to 12 weeks of age was affected by breed, age of the queen, gestation length, delivery by c-section and the presence of birth defects.

## Supplemental Material

sj-docx-1-jfm-10.1177_1098612X241284766 – Supplemental material for Feline dystocia and kitten mortality up to 12 weeks in pedigree cats Appendix 1: Cat breeding questionnaire.

sj-docx-2-jfm-10.1177_1098612X241284766 – Supplemental material for Feline dystocia and kitten mortality up to 12 weeks in pedigree cats Supplemental material, sj-docx-2-jfm-10.1177_1098612X241284766 for Feline dystocia and kitten mortality up to 12 weeks in pedigree cats by Petra Černá, Sneha Joseph Pugalendhi, Darren J Shaw and Danièlle A Gunn-Moore in Journal of Feline Medicine and Surgery

sj-tif-3-jfm-10.1177_1098612X241284766 – Supplemental material for Feline dystocia and kitten mortality up to 12 weeks in pedigree cats Supplementary Figure 1: Relationship between prevalence of birth defects and (a) size of litter, (b) gestation length, (c) age of queen at birth of litter and (d) number of years a breeder has been breeding.

sj-tif-4-jfm-10.1177_1098612X241284766 – Supplemental material for Feline dystocia and kitten mortality up to 12 weeks in pedigree cats Supplementary Figure 2: Relationship between percentage of kittens alive at 12 weeks and (a) age of queen at birth of litter, (b) gestation length, (c) size of litter and (d) number of years a breeder has been breeding.
