# Supplementary material for: Feline dystocia and kitten mortality up to 12 weeks in pedigree cats
Source: J Feline Med Surg. 2024 Dec 10;26(12):1098612X241284766. doi: 10.1177/1098612X241284766 (PMC11632851; doi:10.1177/1098612X241284766)
Supplement: sj-docx-1-jfm-10.1177_1098612X241284766 – Supplemental material for Feline dystocia and kitten mortality up to 12 weeks in pedigree cats [file sj-docx-1-jfm-10.1177_1098612X241284766.docx]

**Cat Breeding Questionnaire**

1. **Your breeding**
2. In which country do you live? ..................
3. Which feline organisation are you registered in?
   1. FIFe  GCCF  TICA  CFA  WCF  ACF  CCCA  NZCF  SACC  Other
4. What breed(s) do you breed? ..................
   1. List of breeds
5. How long have you been breeding for?
   1. .................. years
6. How many queens (female cats) do you have?
   1. 0  1  2  3  4  5  6  7  8  9  10+
7. How many sires (male cats) do you have?
   1. 0  1  2  3  4  5  6  7  8  9  10+
8. How many neutered cats do you have?
   1. 0  1  2  3  4  5  6  7  8  9  10+
9. Do you mainly use your own males for mating, or do you mainly travel for mating?
   1. Mainly own males
   2. Travel for mating
   3. Both
10. How many litters did you have in 2019?
    1. 0  1  2  3  4  5  6  7  8  9  10+
11. **The birth**
12. Number of days from the first mating to the birth (if known)
    1. 61  62  63  64  65  66  67  68  69  70  71  72  73  74+
13. Were you present at the birth?
    1. Yes  No  Some of the time
14. What was the time interval between the first contractions and the birth of the first kitten?
    1. Less than ½ hr  ½-1 h  1-2 h  2-4 h  More than 4 h
15. What was the time interval between the birth of the first kitten and the last kitten?
    1. Less than 6 hr  6-12 h  12-24 h  24-48 h  More than 48 h
16. What was the average time interval between the birth of the kittens?
    1. Less than 1 hr  1–3 h  3–6 h  Other ....................................
17. Did the cat need veterinary assistance?
    1. Yes  No
18. If yes, did she have a c-section?
    1. Yes  No
19. If you answered YES to Q16, what was the reason for the c-section?
    1. Weak contractions
    2. Contractions ended
    3. Kitten(s) were too big
    4. Kitten was obstructed in the birth canal
    5. Dead kitten in the uterus
    6. Uterine torsion (twisted uterus)/rupture
    7. Uterine prolapse
    8. Elective c-section
20. If you answered YES to Q16, did the queen have a c-section in her previous litter?
    1. Yes  No
21. If you answered YES to Q16, were there any complications post c-section?
    1. Queen refused kittens
    2. Queen did not have enough milk
    3. Kitten(s) died
    4. Queen died
    5. Other: please state
    6. No
22. **The litter**
23. Which month was the litter born in? ..................
    1. JAN  FEB  MAR  APR  MAY  JUN  JUL  AUG  SEP  OCT  NOV DEC
24. What breed were the kittens? ...........................................................
25. What colour were the kittens? ..........................................................
26. What age was the queen when the litter was born?
    1. .................. years
27. How many litters did the queen have previously?
    1. 0  1  2  3  4  5  6+
28. How many kittens were born in the litter?
    1. Alive? 0  1  2  3  4  5  6  7  8  9  10+
    2. Dead? 0  1  2  3  4  5  6  7  8  9  10+
29. Birth weights (if known) – please state in grams:
    1. Alive........................................................................................................
    2. Dead .......................................................................................................
30. Were any kittens born with physical defects?
    1. Yes  No
31. If you answered YES on Q26 please provide details on what type defect your kittens had and how many kittens in the litter were affected? If you can also provide a picture, that would be very helpful.
32. Number of kittens alive after:
    1. 1 week 0  1  2  3  4  5  6  7  8  9  10+
    2. 8 weeks 0  1  2  3  4  5  6  7  8  9  10+
    3. 12 weeks 0  1  2  3  4  5  6  7  8  9  10+
33. If any of the kittens died in those weeks, can you please provide details on the cause(s) of death?
    1. 1 week:
    2. 8 weeks:
    3. 12 weeks:
