# Supplementary material for: Feline dystocia and kitten mortality up to 12 weeks in pedigree cats
Source: J Feline Med Surg. 2024 Dec 10;26(12):1098612X241284766. doi: 10.1177/1098612X241284766 (PMC11632851; doi:10.1177/1098612X241284766)
Supplement: sj-docx-2-jfm-10.1177_1098612X241284766 – Supplemental material for Feline dystocia and kitten mortality up to 12 weeks in pedigree cats [file sj-docx-2-jfm-10.1177_1098612X241284766.docx]

**Supplementary Figure 1** - Relationship between prevalence of birth defects and A) Size of litter; B) Gestation length; C) Age of queen at birth of litter; and D) Number of years a breeder has been breeding. Size of points are scaled to the number of litters at a particular x-value. Vertical lines are the 95% confidence limits for each prevalence value. For A,C, D) solid lines and associated shaded area are fitted linear binomial general linear model fits and standard errors. For B) solid line and associated shaded area is a LOESS running average and standard error.

**Supplementary Figure 2** - Relationship between percentage of kittens alive at 12 weeks and A) Age of queen at birth of litter; B); Gestation length C) Size of litter; and D) Number of years a breeder has been breeding. Size of points are scaled to the number of litters at a particular x-value. Vertical lines are the 95% confidence limits for each prevalence value. For A, D) solid lines and associated shaded area are fitted linear binomial general linear model fit and standard errors. For B,C) solid line and associated shaded area are fitted polynomial binomial general linear model fit and standard errors.

**Appendix 1**: Cat breeding Questionnaire
