# Supplementary figures and images for: Feline dystocia and kitten mortality up to 12 weeks in pedigree cats
Source: J Feline Med Surg. 2024 Dec 10;26(12):1098612X241284766. doi: 10.1177/1098612X241284766 (PMC11632851; doi:10.1177/1098612X241284766)

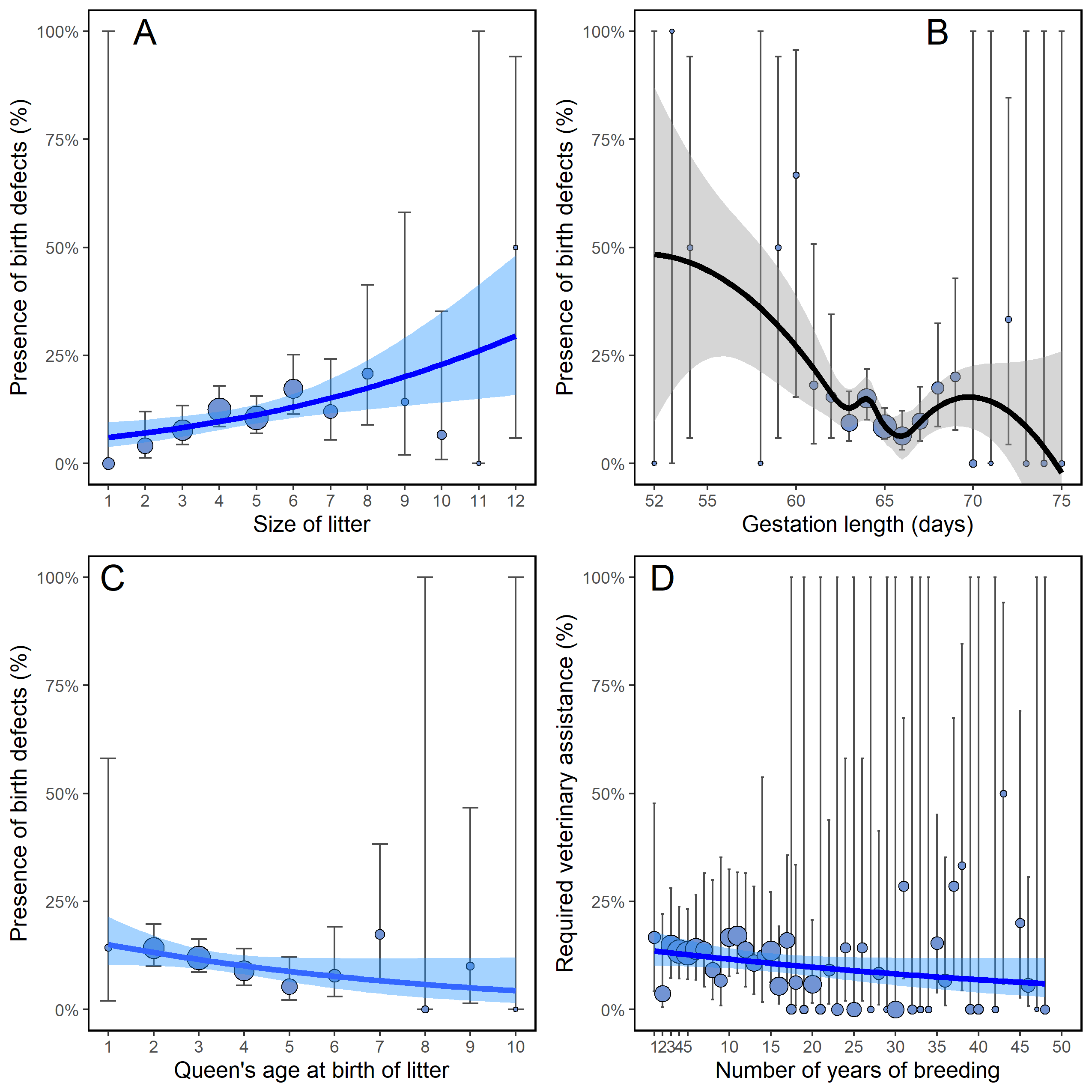

Supplement: sj-tif-3-jfm-10.1177_1098612X241284766 – Supplemental material for Feline dystocia and kitten mortality up to 12 weeks in pedigree cats [file sj-tif-3-jfm-10.1177_1098612X241284766.tif]

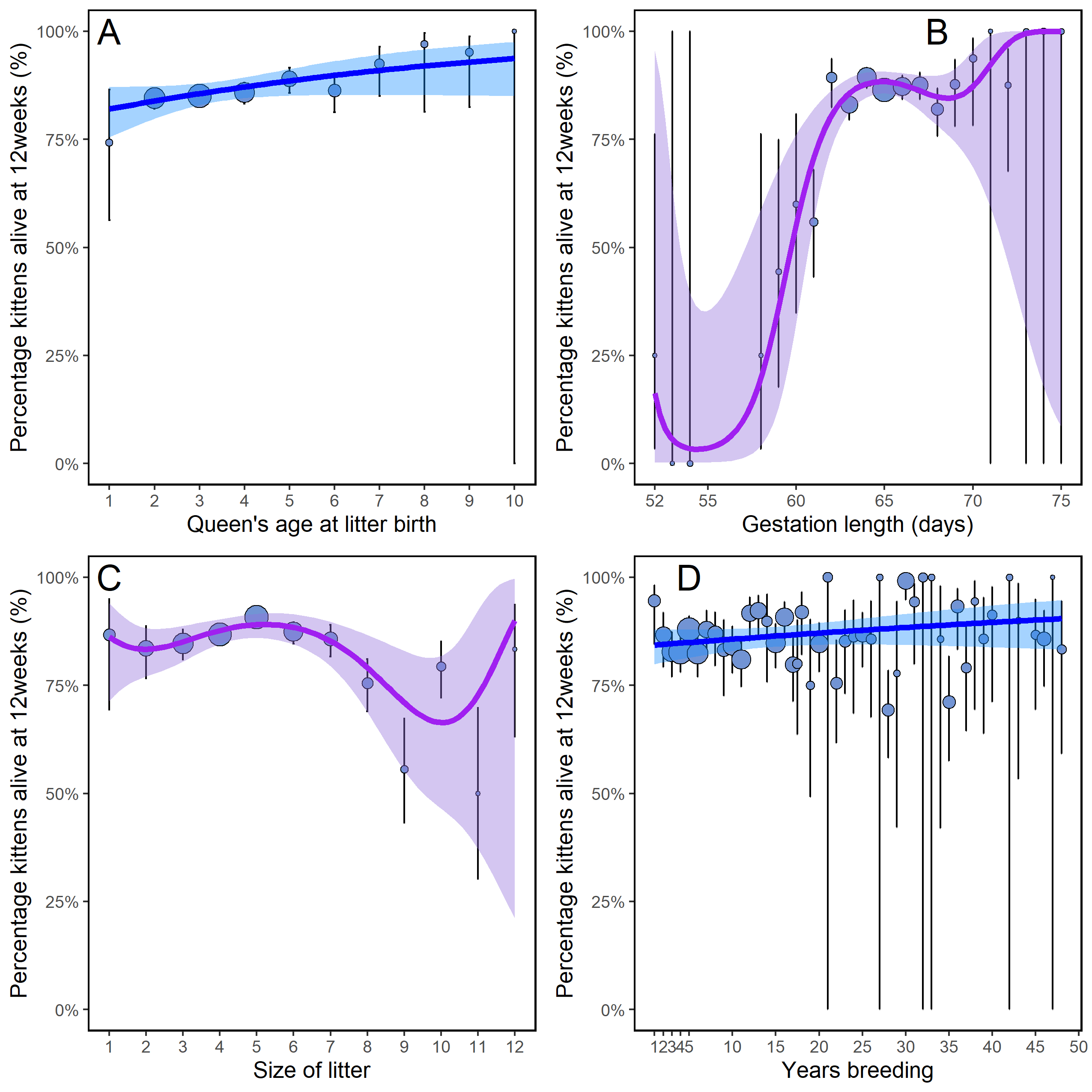

Supplement: sj-tif-4-jfm-10.1177_1098612X241284766 – Supplemental material for Feline dystocia and kitten mortality up to 12 weeks in pedigree cats [file sj-tif-4-jfm-10.1177_1098612X241284766.tif]
